# How I Do It: Middle Fossa Approach of Facial Nerve Decompression

**DOI:** 10.1002/lary.70210

**Published:** 2025-10-17

**Authors:** Stéphane Gargula, Ralph Haddad, Dario Ebode, Maria‐Pia Tuset, Justin Michel, Thomas Radulesco, Lucas Troude, Mary Daval

**Affiliations:** ^1^ ENT‐HNS Department Aix Marseille Univ, APHM, CNRS, IUSTI, La Conception University Hospital Marseille France; ^2^ ENT‐HNS Department Hospital Fondation Adolphe de Rothschild Paris France; ^3^ Aix Marseille University, APHM, Neurosurgery Department North University Hospital, Chemin des Bourrely Marseille France

**Keywords:** decompression, facial nerve, facial paralysis, skull base, surgical

## Abstract

We present a reproducible, stepwise middle fossa approach for facial nerve decompression focused on the labyrinthine segment, geniculate ganglion, and meatal foramen, with consistent anatomical landmarks to preserve hearing. The article and video detail patient setup, safe corridor creation, and retrograde drilling with practical tips to avoid cochlear or semicircular canal injury, aiming to lower the learning curve for facial nerve decompression.

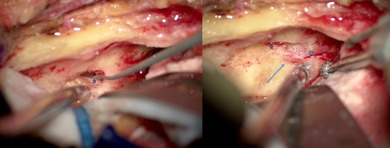

## 1 Introduction

Severe peripheral facial paralysis can significantly impair quality of life [1]. Surgical decompression may be indicated in cases (particularly traumatic) with poor electrophysiological prognosis [2]. Most authors agree this evaluation is more reliable after several weeks and that surgery should be performed within 2 months. Lesions mostly involve the peri‐geniculate region, requiring decompression of the labyrinthine segment and meatal foramen [1, 3, 4, 5]. The middle fossa approach remains the technique of choice when hearing preservation is essential [1, 4]. Although radiosurgical advances reduced its use for vestibular schwannomas, the approach remains essential for facial nerve decompression [1, 4, 5]. We describe a stepwise method using consistent anatomical landmarks, designed to make this demanding approach more accessible and accommodating anatomical variations.

## 2 Materials and Methods

We describe the step‐by‐step technique with illustrative surgical footage (Video 1).

**VIDEO 1:** Commented surgical footage. Video content can be viewed at https://onlinelibrary.wiley.com/doi/10.1002/lary.70210.

## 3 Results

Under general anesthesia, the patient is positioned supine with the head rotated contralaterally. Intracranial pressure is optimized with hyperventilation (End‐tidal CO_2_ target 28–30 mmHg) and reverse Trendelenburg in coordination with anesthesia. Hyperosmotic agents may be used on a case‐by‐case basis (e.g., 500 cc of mannitol if intracranial pressure interferes with the surgical procedure). A single dose of intravenous dexamethasone (8 mg IV) and antibiotic prophylaxis are systematically administered at induction, typically using cefazolin (2 g IV) or amoxicillin‐clavulanic acid (2 g/200 mg IV), depending on local protocol and patient allergy status. Facial nerve monitoring is established using a two‐channel system, followed by antiseptic preparation and sterile draping. A pretragal incision is extended upward into the temporal scalp (~7 cm). Temporalis fascia is harvested, and the muscle is split vertically with a transverse cut to expose the squama and zygomatic root. A 4 × 4 cm craniotomy is performed over the zygomatic root. Depending on the hairline, a slightly convex incision anteriorly helps avoid the common pitfall of an overly posterior bone flap The external auditory meatus should be projected at the junction of the posterior third and anterior two‐thirds of the bone flap. Dural tears may occur when lifting the bone flap or drilling. In such situations, CSF may be allowed to drain to release pressure, and therefore wait until the end of the procedure to close the tear. It may also be decided to make a small dural incision for this purpose.

Before beginning to detach the middle fossa floor, it is preferable to adjust the lower edge of the craniotomy to the floor level and smooth the edges, then detach the dura circumferentially to limit tension. The anterolateral portion of the temporal bone is highly variable, and there may be significant inclination at the anterior part of the exposure. Preoperative CT helps anticipate anatomical variations. Gentle cauterization of the temporal dura retracts it and significantly improves exposure. Venous bleeding often occurs at the anterior part of the exposure and can be controlled using bipolar forceps, bone wax, compression, etc.

**FIGURE 1:**
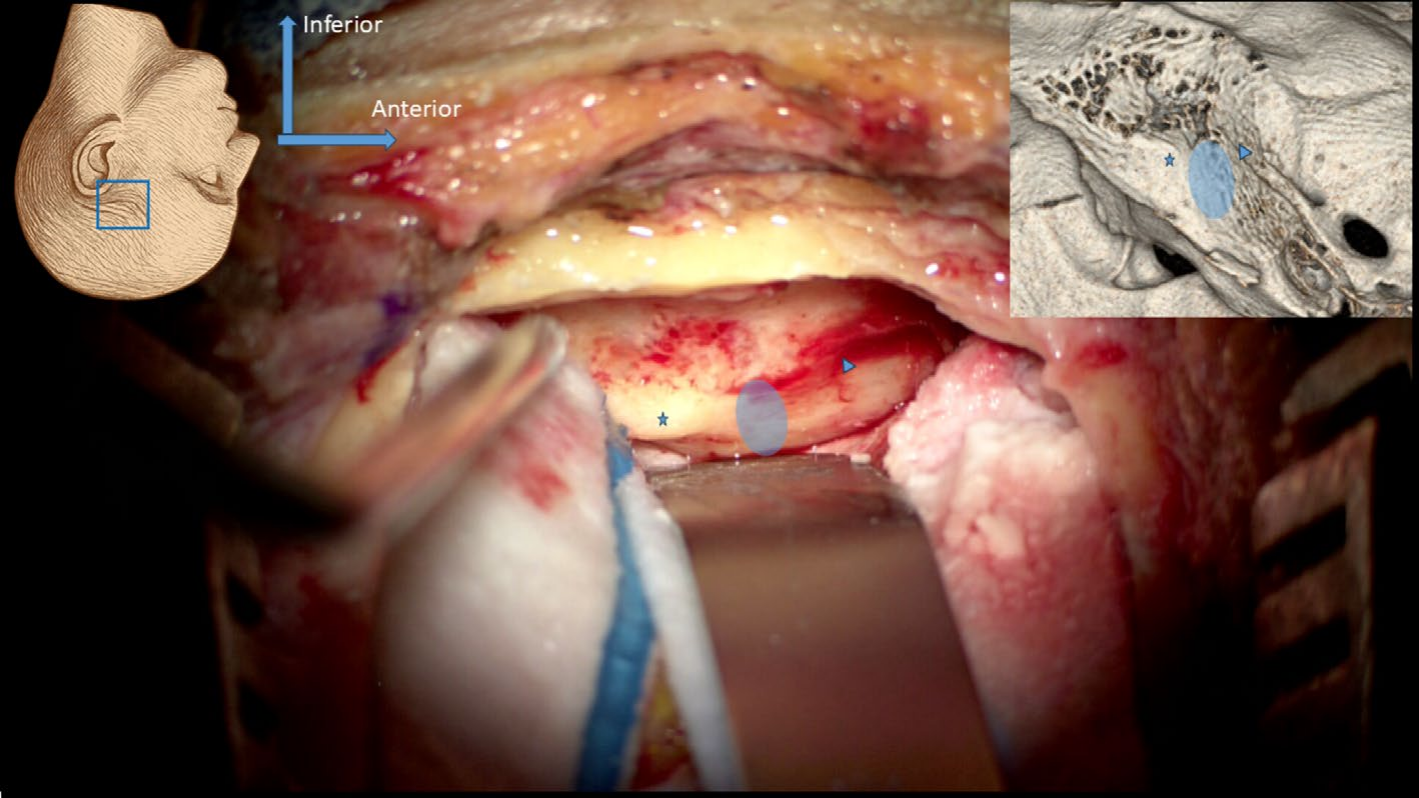
Facial nerve exposure landmarks. SSCC (starred); petrosal nerves (arrowhead); projected drilling area (ellipse).

The use of neurosurgical patties at the anterior and posterior ends of the elevation allows the dura to be retracted without the use of a retractor at these early stages. Tegmen mastoideum and antri are usually distinguishable by trabeculations and translucency. The elevation of the dura must then be carried out starting from the posterior part up to its limit, the petrous ridge, where the superior petrosal sinus can be visualized. The dissection continues anteriorly along the axis of the greater and lesser petrosal nerves, in order to separate them from the dura without causing trauma. In some cases the geniculate ganglion may be dehiscent, which can be anticipated on the preoperative CT‐scan. Transection of the middle meningeal artery is usually unnecessary. Ideally, the bone flap should be centered on the meatal plane, which will be the region bisecting the axis of the arcuate eminence and the petrosal nerves (Figure 1). If this is not the case, additional drilling should be performed before proceeding to the next steps. For the following steps, retractors (e.g., Yasargil, House‐Urban) aid in stabilizing exposure.

**FIGURE 2:**
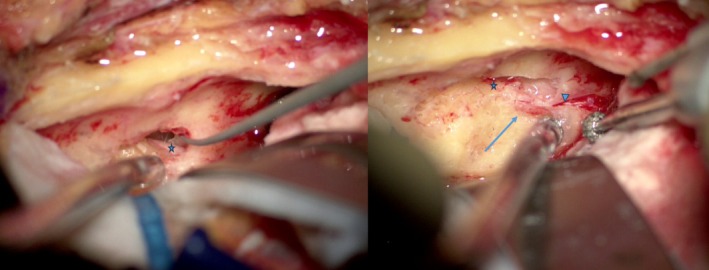
Retrograde exposure of the geniculate ganglion (right panel, arrow) from the tympanic segment (starred) and the petrosal nerves (right panel, arrowhead).

The middle ear is opened above the tegmen antri, with a careful anterior extension to expose the ossicular chain. The main danger of this step is injury to the ossicular chain and the resulting trauma to the inner ear. The lateral semicircular canal and the tympanic segment of the facial nerve are identified. Intraoperative monitoring generally remains silent for cases of complete preoperative paralysis. Drilling follows the “eggshell” technique, progressing retrograde from the tympanic segment and petrosal nerves to the geniculate ganglion. (Figure 2).

**FIGURE 3:**
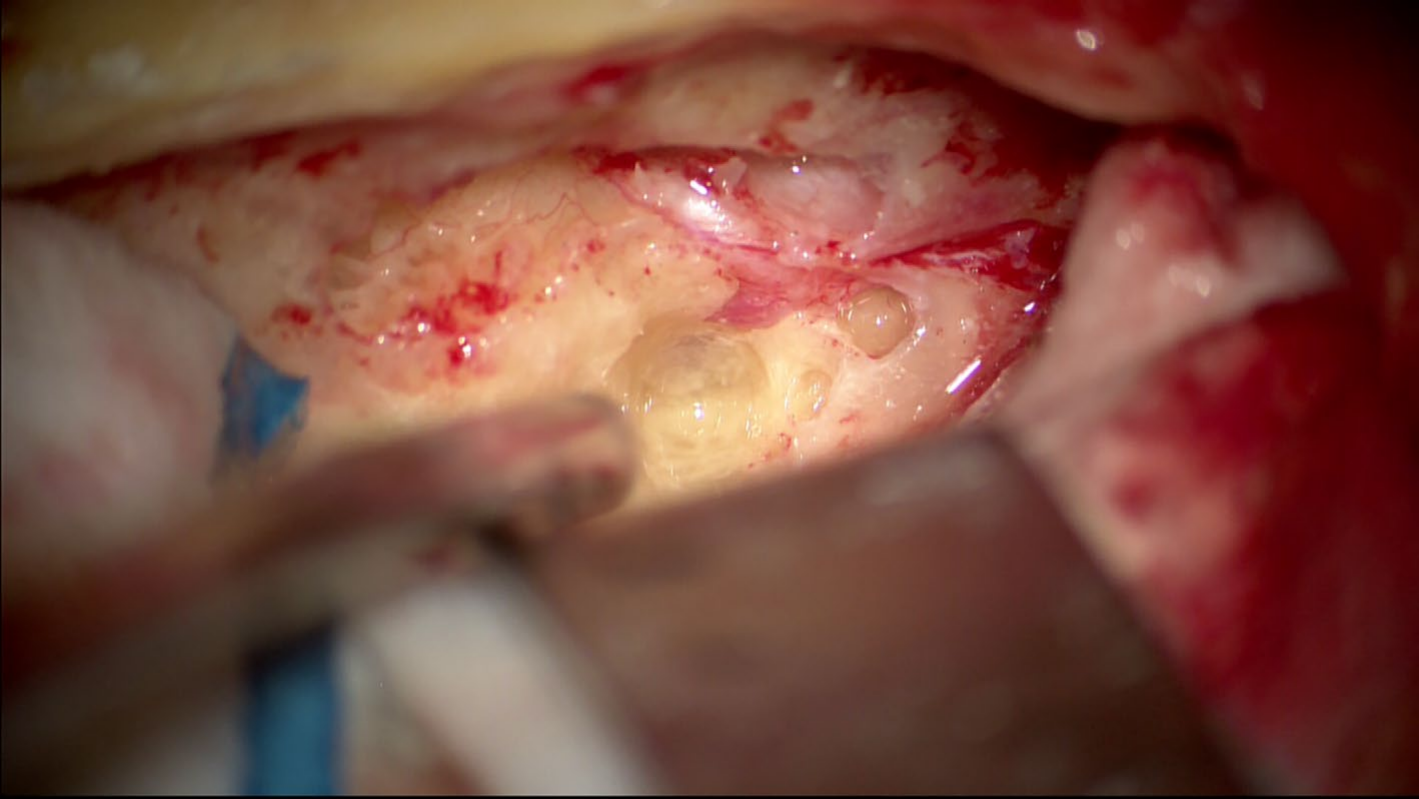
Region at risk for cochlear damage (danger sign); facial nerve (blue star).

**FIGURE 4:**
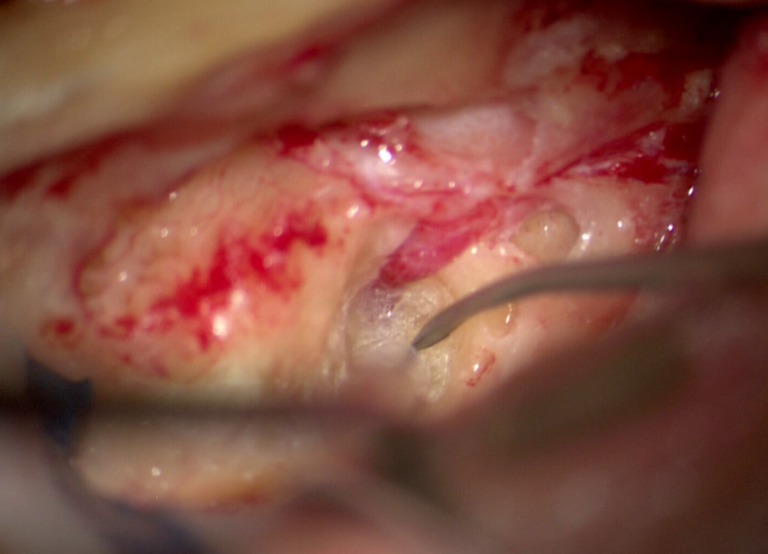
Exposure of the meatal foramen.

Drilling the trabecular bone surrounding the superior semicircular canal (SSCC) is necessary in most cases to provide sufficient space for dissection of the geniculate ganglion and prepare for the dissection of the labyrinthine portion of the facial nerve. The lateral semicircular canal is a good reference point for its level. In the case of an unfavorable anatomical situation with a deeply located first portion, it is preferable to “blue‐line” the SSCC in order to get as close as possible to it and increase the space available. The width of the operative corridor between the cochlear apex and SSCC can be anticipated. The primary risk of dissecting the labyrinthine portion of the facial nerve is breaching the cochlear apex, which may result in irreversible hearing loss (Figure 3). Drilling continues along the axis of the internal auditory canal, avoiding drilling anteriorly and inferiorly to the labyrinthine segment, which must be decompressed on its posterosuperior portion. The dissection continues to the fundus, which must be clearly identified, with release of the meatal foramen (Figure 4). Some authors recommend incision of the dura mater and arachnoid membrane of the meatal foramen. At the end of the procedure, check that the tympanic segment and the emergence of the petrosal nerves are also properly decompressed.

Reconstruction of the tegmen defect is achieved using a bone fragment from the craniotomy, covered with temporalis fascia to ensure watertight closure. The rest of the bone flap can be replaced with sutures or plates, with one or more suspension points of the dura. The muscle will be sutured with absorbable sutures and the skin incision according to the surgeon's usual practice. A compressive dressing is applied.

Neither corticosteroids nor antibiotics are continued postoperatively on a routine basis. Early postoperative monitoring includes neurological observation and a high‐resolution temporal bone CT scan within 24 h to assess bone flap positioning, middle fossa reconstruction, and potential complications (although very rare, extradural hematoma). No preoperative vaccination is performed due to the extremely low risk of postoperative CSF leak.

## 4 Discussion

The extent of decompression needed to achieve a satisfactory result remains difficult to define as no controlled studies have compared “partial” versus “complete” decompression. In clinical practice, it appears reasonable to decompress the entire segments of the nerve involved in the lesion mechanism which are surgically accessible, provided this can be done without endangering adjacent structures such as the cochlea, semicircular canals, or neurological elements. Few comparative studies have rigorously assessed the benefit of different methods of surgical decompression versus conservative management in traumatic or idiopathic facial paralysis. The main adverse prognostic factor in facial paralysis is the loss of macroscopic integrity of the facial nerve: when a complete or partial transection is confirmed (or required because of lesion or tumor), surgical exposure and often nerve grafting are required, making optimal anatomical access critical [1, 4]. While the middle fossa approach offers unparalleled exposure of the labyrinthine and geniculate segments and preserves hearing, it entails specific risks—most notably epidural hematoma, which requires close postoperative monitoring and may justify initial management in an intensive‐care unit. These risks must be carefully balanced against the substantial functional and psychosocial burden of peripheral facial palsy. Although technically demanding, the stepwise method we describe may help reduce barriers to its adoption among surgeons less familiar with this route.

## Ethics Statement

The patient provided consent for the use of surgical footage and imaging.

## Conflicts of Interest

The authors declare no conflicts of interest.

## Data Availability

Data sharing not applicable to this article as no datasets were generated or analysed during the current study.
